# Crystal and mol­ecular structures of some phosphane-substituted cymantrenes [(C_5_H_4_ *X*)Mn(CO)*LL*′] (*X* = H or Cl, *L* = CO, *L*′ = PPh_3_ or PCy_3_, and *LL*’ = Ph_2_PCH_2_CH_2_PPh_2_)

**DOI:** 10.1107/S2053229621009177

**Published:** 2021-09-27

**Authors:** Karlheinz Sünkel, Christian Klein-Hessling

**Affiliations:** a Chemistry, Ludwig-Maximilians-University Munich, Butenandtstrasse 9, Munich, D-81377, Germany

**Keywords:** cymantrene, irradiation, phosphane com­plexes, crystal structure, weak inter­actions

## Abstract

UV irradiation of tetra­hydro­furan solutions of [CpMn(CO)_3_] (Cp = π-C_5_H_5_ or π-C_5_H_4_Cl) in the presence of the phosphanes PPh_3_ or PCy_3_ (Cy = cyclo­hex­yl) and Ph_2_PCH_2_CH_2_PPh_2_ yields the substitution products [CpMn(CO)_2_P*R*_3_] (*R* = Ph or Cy) and [CpMn(CO)(Ph_2_PCH_2_CH_2_PPh_2_)], namely, dicarbon­yl(η^5^-cyclo­penta­dien­yl)(tri­phenyl­phosphane-κ*P*)manganese(I), [Mn(C_5_H_5_)(C_18_H_15_P)(CO)_2_], **1a**, dicarbon­yl(η^5^-1-chloro­cyclo­penta­dien­yl)(tri­phenyl­phosphane-κ*P*)man­ganese(I), [Mn(C_5_H_4_Cl)(C_18_H_15_P)(CO)_2_], **1b**, dicarbon­yl(η^5^-cyclo­penta­dien­yl)(tri­cyclo­hexyl­phosphane-κ*P*)manganese(I), [Mn(C_5_H_5_)(C_18_H_33_P)(CO)_2_], **2a**, di­carbon­yl(η^5^-1-chloro­cyclo­penta­dien­yl)(tri­cyclo­hexyl­phosphane-κ*P*)manganese(I), [Mn(C_5_H_4_Cl)(C_18_H_33_P)(CO)_2_], **2b**, carbon­yl(η^5^-cyclo­penta­dien­yl)[1,2-bis­(di­phenyl­phosphan­yl)ethane-κ^2^ *P*,*P*′]manganese(I), [Mn(C_5_H_5_)(C_26_H_24_P_2_)(CO)], **3a**, and carbon­yl(η^5^-1-chloro­cyclo­penta­dien­yl)[1,2-bis­(di­phenyl­phosphan­yl)ethane-κ^2^ *P*,*P*′]manganese(I), [Mn(C_5_H_4_Cl)(C_26_H_24_P_2_)(CO)], **3b**, The crystal structure determinations show a very small influence of the chlorine substitution and a moderate influence of the phosphane substitution on the bond lengths. The P*R*_3_ groups avoid being eclipsed with the C—Cl bonds. All the com­pounds employ weak C—H⋯O inter­actions for inter­molecular association, which are enhanced by C—H⋯Cl contacts in the chlorinated products.

## Abstract 2

The syntheses and structures of the cymantrenes [(C_5_H_4_ *X*)Mn(CO)*LL*′] (*X* = H or Cl; *L* = CO: *L*′ = PPh_3_ or PCy_3_; *LL*′ = Ph_2_PCH_2_CH_2_PPh_2_) are reported. Substitution of CO by phosphanes influences the bond parameters more than replacing the C_5_H_5_ ligand by C_5_H_4_Cl.

## Introduction   

The substitution of carbon monoxide (CO) by other donor ligands, particularly phosphanes, is one of the most important textbook examples for the reactivity of metal carbonyl com­plexes (Elschenbroich, 2016 ▸; Crabtree, 2005 ▸; Jordan, 2007 ▸). This is also true for the so-called ‘piano-stool’ com­plexes, which contain, besides CO ligands, aromatic π-ligands like benzene or the cyclo­penta­dienyl anion. Many studies have shown that the nature of the π-ligand strongly influences the ease of CO substitution (Veiros, 2000 ▸; Le Moigne *et al.*, 1976 ▸). But *vice versa*, the aromatic reactivity depends also on the electronic situation within the metal carbonyl moiety (Fan & Hall, 2001 ▸). One of the most studied systems is the ‘cymantrene’ series CpMn(CO)_3_ and its substituted derivatives (Caulton, 1981 ▸). The substitution of one or two CO ligands by mono- or bidentate phosphanes was studied in the 1960s and it was found that the best way to do this was by UV irradiation (Strohmeier & Barbeau, 1962 ▸; Nyholm *et al.*, 1963 ▸; Khatami *et al.*, 1972*a* ▸; Kursanov *et al.*, 1970 ▸; Young & Wrighton, 1989 ▸). The choice of solvent and the irradiation time were the main determinants for the formation of either mono- or disubstitution products. Later on, studies on the spectroscopic [IR, ESR (electron spin resonance) and NMR] (Rehder & Keçeci, 1985 ▸; Ginzburg *et al.*, 1974 ▸; Pike *et al.*, 1989 ▸) and electrochemical properties (Treichel *et al.*, 1975 ▸; Connelly & Kitchen, 1977 ▸) followed, which showed, as might have been expected, that the introduction of aryl- or alkylphosphanes led to increased electron density at the metal. Further studies were devoted to the reactivity in protonation reactions (Ginzburg *et al.*, 1974 ▸), electrophilic hydrogen exchange reactions (Setkina *et al.*, 1973 ▸; Khatami *et al.*, 1972*b* ▸; Antonova & Shapiro, 1991 ▸) and deprotonation by butyl lithium (Loim *et al.*, 1988 ▸). A survey of the Cambridge Structural Database (CSD, Version 5.42, accessed on 26th August, 2021; Groom *et al.*, 2016 ▸) showed no crystal structures for the fragments [(C_5_H_4_Cl)Mn(CO)P] and about 80 entries for the corresponding C_5_H_5_-containing fragments. Limitation of the search to the fragment [(C_5_H_5_)Mn(CO)_2_PPh_2_] gave 10 hits, of which most contained unsymmetrical mono- or dinuclear diphos­phanes. Relevant in the context of this study were an early determination of the structure of [(C_5_H_5_)Mn(CO)_2_(PPh_3_)] (Barbeau *et al.*, 1972 ▸) and the crystal structure of [(C_5_H_5_)Mn(CO)_2_PPh_2_CH_2_Ph] (CSD refcode GIXRIO; Geicke *et al.*, 1998 ▸). No hits were obtained for chelating diphosphanes, except for a derivative of 1,1′-bis­diphenyl­phos­phanyl­ferro­cene (EFUHAO; André-Ben­tabet *et al.*, 2002 ▸). We felt it might contribute to a better understanding of this substance class to add some more crystal structure determinations.
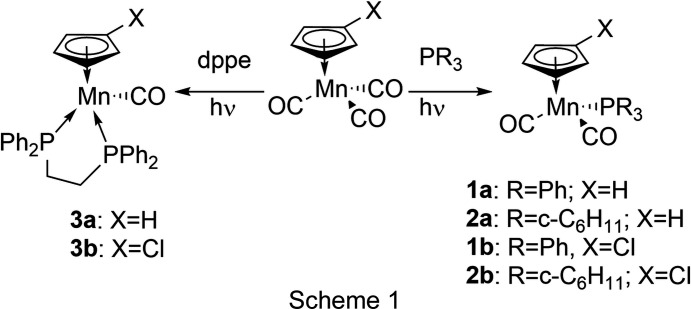


## Experimental   

### Synthesis and crystallization   

The syntheses of com­pounds **1a**, **1b**, **2a** and **3a** have been described previously (Strohmeier & Barbeau, 1962 ▸; Khatami *et al.*, 1972*a* ▸; Strohmeier & Müller, 1967 ▸; Nyholm *et al.*, 1963 ▸).

#### General procedure for the synthesis of 1a, 2a and 3a   

A solution of [(C_5_H_5_)Mn(CO)_3_] (**I**) and a slight molar excess of the phosphane in tetra­hydro­furan (THF, 120 ml) was irradiated for 7 h under argon. The colours of the solutions changed from yellow to red with concomitant gas evolution. After further stirring for 16 h, the solvent was evacuated and the residue dissolved in diethyl ether (Et_2_O) and filtered through a plug of silica gel. The solvent was evaporated again and the residue dissolved in the minimum amount of petroleum ether. This solution was placed on top of a silica gel chromatography column (alumina in the case of **3a**) and the products were eluted with a petroleum ether/Et_2_O (9:1 *v*/*v*) mixture. Evaporation of the eluate yielded the products as yellow powders. Recrystallization from petroleum ether (with some added Et_2_O) by slow evaporation in a refrigerator at 5 °C yielded crystals of all three com­pounds.

Compound **1a** was prepared from **I** (3.00 g, 14.7 mmol) and PPh_3_ (4.20 g, 16.0 mmol) in a yield of 3.90 g (8.9 ± 0.01 mmol, 61%). 0.57 g of com­pound **I** (2.8 ± 0.01 mmol, 19%) were recovered. ^1^H NMR (CDCl_3_, 400 MHz): δ 7.57–7.20 (*m*, 15H), 4.31 (*s*, 5H). ^13^C{^1^H} NMR (CDCl_3_, 101 MHz): δ 232.8 (*d*, *J* = 26.8 Hz), 138.2 (*d*, *J* = 40.3 Hz), 133.0 (*d*, *J* = 10.6 Hz), 129.6 (*d*, *J* = 2.4 Hz), 128.2 (*d*, *J* = 9.4 Hz), 82.7. ^31^P{^1^H} NMR (CDCl_3_, 162 MHz): δ 93.1.

Compound **2a** was prepared from **I** (0.78 g, 3.8 mmol) and PCy_3_ (1.12 g, 4.0 mmol) in a yield of 0.11 g (0.24 ± 0.01 mmol, 6%). 0.35 g of com­pound **I** (1.7 mmol, 45%) were recovered. ^1^H NMR (CDCl_3_, 400 MHz): δ 4.48 (*s*, 5H), 2.01–1.11 (*m*, 33H). ^31^P{^1^H} NMR (CDCl_3_, 162 MHz): δ 92.8.

Compound **3a** was prepared from **I** (0.20 g, 1.0 mmol) and 1,2-bis(diphenylphosphanyl)ethane (dppe; 0.44 g, 1.0 mmol) in a yield of 0.15 g (0.27 ± 0.01 mmol, 27%). 0.09 g of com­pound **I** (0.4 ± 0.01 mmol, 45%) were recovered. ^1^H NMR (CDCl_3_, 400 MHz): δ 7.79–7.70 (*m*), 7.44–7.34 (*m*), 7.20–7.11 (*m*), 4.13 (*s*), 2.54–2.41 (*m*), 2.36–2.21 (*m*). ^31^P{^1^H} NMR (CDCl_3_, 162 MHz): δ 118.6.

#### Synthesis of 1b   

Compound **1b** was prepared according to the method reported by Klein-Hessling *et al.* (2021 ▸). Recrystallization from petroleum ether (with some added Et_2_O) by slow evaporation in a refrigerator at 5 °C yielded crystals. ^1^H NMR (CDCl_3_, 400 MHz): δ 7.49–7.35 (*m*, 15H), 4.48 (*q*, *J* = 2.0 Hz, 2H), 4.00 (*q*, *J* = 2.3 Hz, 2H). ^13^C{^1^H} NMR (CDCl_3_, 101 MHz): δ 231.8 (*d*, *J* = 23.5 Hz), 137.7 (*d*, *J* = 41.2 Hz), 133.0 (*d*, *J* = 10.5 Hz), 129.8, 128.4 (*d*, *J* = 9.6 Hz), 101.3, 81.5, 81.0. ^31^P{^1^H} NMR (CDCl_3_, 162 MHz): δ 91.8.

#### Synthesis of 2b   

A solution of impure [(C_5_H_4_Cl)Mn(CO)_3_] (0.50 g, purity *ca* 92%) and PCy_3_ (0.90 g, 3.2 ± 0.01 mmol) in THF (120 ml) was irradiated for 7 h. After the usual work up (see above), a yellow solid was obtained, consisting of a 7:3 mixture of **2b** and **2a**. Recrystallization from petroleum ether (with some added Et_2_O) by slow evaporation in a refrigerator at 5 °C yielded crystals. ^1^H NMR (CDCl_3_, 270 MHz): δ 4.63 (2H), 4.33 (2H), 2.02–1.07 (33H). ^31^P{^1^H} NMR (CDCl_3_, 162 MHz): δ 91.8. MS (EI, 70 eV): *m*/*z* = 490.4 (*M* ^+^), 434.4 (*M* ^+^ − 2CO).

#### Synthesis of 3b   

A solution of [(C_5_H_4_Cl)Mn(CO)_3_] (0.35 g, 1.5 ± 0.01 mmol) and dppe (0.62 g, 1.55 ± 0.01 mmol) in THF (120 ml) was irradiated for 7 h. After usual work up, **3b** (0.34 g, 0.6 ± 0.01 mmol, 40%) was isolated as an orange powder. 0.05 g of the starting material (0.25 ± 0.01 mmol, 17%) was recovered. Recrystallization from petroleum ether (with some added Et_2_O) by slow evaporation in a refrigerator at 5 °C yielded crystals. ^1^H NMR (CDCl_3_, 400 MHz): δ 7.82–7.75 (4H), 7.47–7.35 (6H), 7.33–7.22 (6H), 7.19–7.09 (4H), 4.44 (2H), 3.56 (2H), 2.53–2.41 (2H), 2.36–2.22 (2H). ^13^C{^1^H} NMR (CD_2_Cl_2_, 101 MHz): δ 232.8 (*t*, *J* = 25.2 Hz), 142.9 (*dt*, *J* = 22.4, 11.4 Hz), 139.83–138.89 (*m*), 133.1 (*t*, *J* = 5.4 Hz), 131.4 (*t*, *J* = 4.7 Hz), 129.4, 128.6, 128.1 (*dt*, *J* = 8.7, 4.4 Hz), 97.5, 78.0, 77.9, 77.6, 30.6 (*t*, *J* = 21.2 Hz). ^31^P{^1^H} NMR (CDCl_3_, 162 MHz): δ 117.6. IR (KBr, cm^−1^): *ν* (CO) = 1847. MS (EI, 70 eV): *m*/*z* = 580.3 (*M* ^+^), 552.3 (*M* ^+^ − CO), 398.2 (C_26_H_24_P_2_), 183.0 (PPh_2_), 108.0 (PPh). HRMS (EI): *m*/*z* calculated 580.0684, found: 580.0681 (*M* ^+^).

### Refinement   

In the refinements of **2a** and **2b**, a rigid-body restraint was used for the C3—C4 and C2—C3 bonds, respectively, because they had failed the ‘Hirshfeld-Test’ of *PLATON* (Spek, 2020 ▸) significantly. All H atoms were constrained. For com­pound **3a**, *PLATON* analysis showed 16% solvent-accessible voids. Therefore, the SQUEEZE program (Spek, 2015 ▸) was used, which recovered 221 e per unit cell. Crystal data, data collection and structure refinement details are summarized in Table 1 ▸.

For the discussion of hydrogen bonds, the standard settings of *Mercury* (Macrae *et al.*, 2020 ▸) (H atoms present, *D*—H⋯*A* angle > 120.0°, ‘all donors’, contact distance range ‘sum of vdW radii minus 5.00 to sum of vdW radii plus 0.00’) were used for all com­pounds except **1b**, where the ‘sum of vdW radii plus 0.10’ was used as the upper limit.

## Results and discussion   

### [(C_5_H_4_ *X*)Mn(CO)_2_(PPh_3_)], *X* = H (1a) and Cl (1b)   

Both **1a** and **1b** have been known for some time (Strohmeier & Barbeau, 1962 ▸; Khatami *et al.*, 1972*a* ▸,*b* ▸; Kursanov *et al.*, 1970 ▸; Barbeau *et al.*, 1972 ▸) and were prepared by irradiation of the corresponding tri­carbonyls in the presence of PPh_3_. Deprotonation of **1a** with butyl lithium, followed by electronic quenching with C_2_Cl_6_, yielded **1b** (Klein-Hessling *et al.*, 2021 ▸). A crystal structure determination of **1a** had been reported nearly 50 years ago (Barbeau *et al.* 1972 ▸). That com­pound was crystallized from benzene/ethanol in the triclinic space group *P* 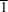.

Irradiation of THF solutions of [(C_5_H_4_ *X*)Mn(CO)_3_] in the presence of PPh_3_ leads to **1a** and **1b** in moderate yields of 40–60% (Scheme 1). Substantial amounts of the starting materials could be recovered. Products were isolated by chromatography and recrystallized from petroleum ether/Et_2_O.

#### Mol­ecular and crystal structure of 1a   

**Figure 1:**
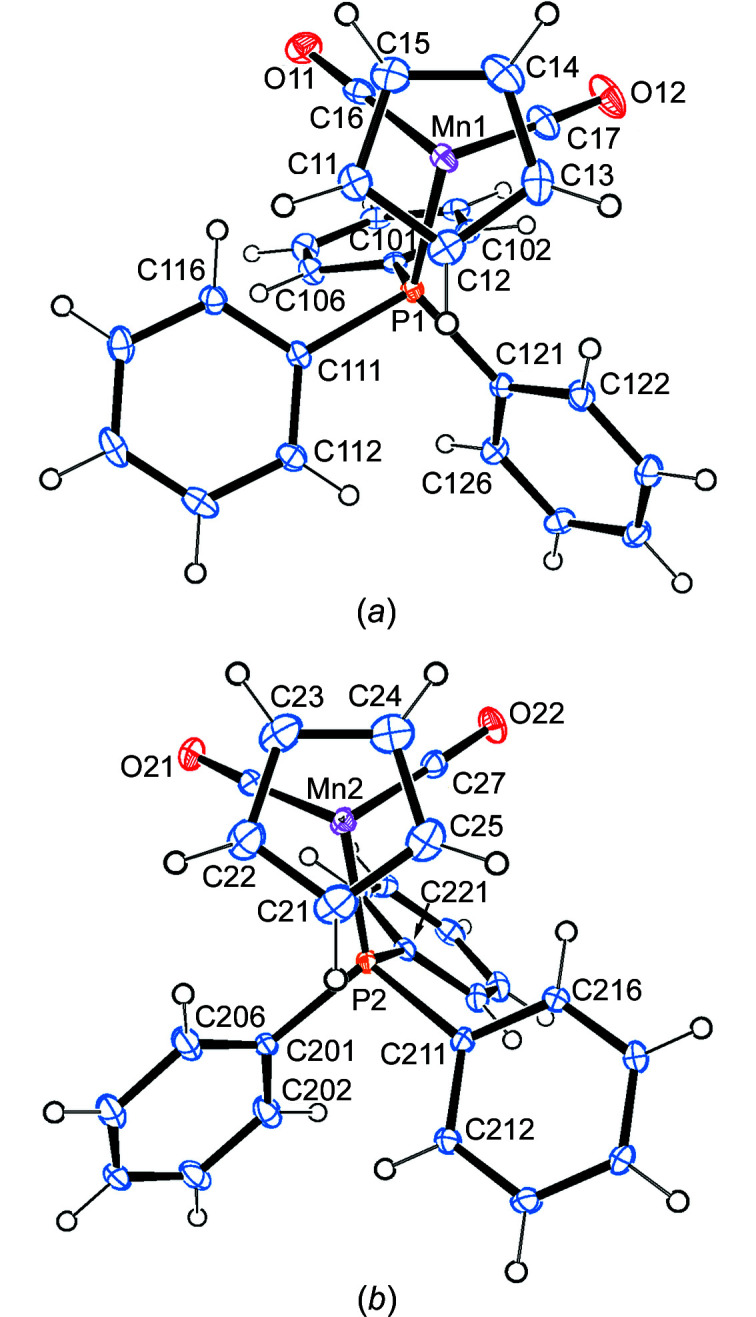
The molecular structures of (*a*) mol­ecule *A* and (*b*) mol­ecule *B* of com­pound **1a**, with displacement ellipsoids drawn at the 30% probability level.

The crystals of **1a** obtained from petroleum ether/Et_2_O are apparently a different modification than those described in the literature. Our com­pound crystallized in the monoclinic space group *P*2_1_/*n* with two independent mol­ecules in the asymmetric unit (Fig. 1 ▸).

The major difference between the two mol­ecules is in the relative orientation of the Mn(CO)_2_P tripod and the projection of the cyclo­penta­dienyl ring. While in mol­ecule *A* both Mn→P and one Mn→CO vector nearly eclipse C—H bonds of the cyclo­penta­dienyl ring, in mol­ecule *B* this is the case for the Mn→P vector only. In addition, the Mn2—P2 bond [2.2421 (7) Å] is significantly longer (>20σ) than the Mn1—P1 bond [2.2259 (6) Å]. All other bond lengths are identical in the two mol­ecules (Table 2 ▸).

There are five inter­molecular C—H⋯O hydrogen bonds (Table S1 in the supporting information). Three of them involve arene C—H bonds, and carbonyl atom O22 accepts two of them (Fig. S1).

#### Mol­ecular structure of 1b   

**Figure 2:**
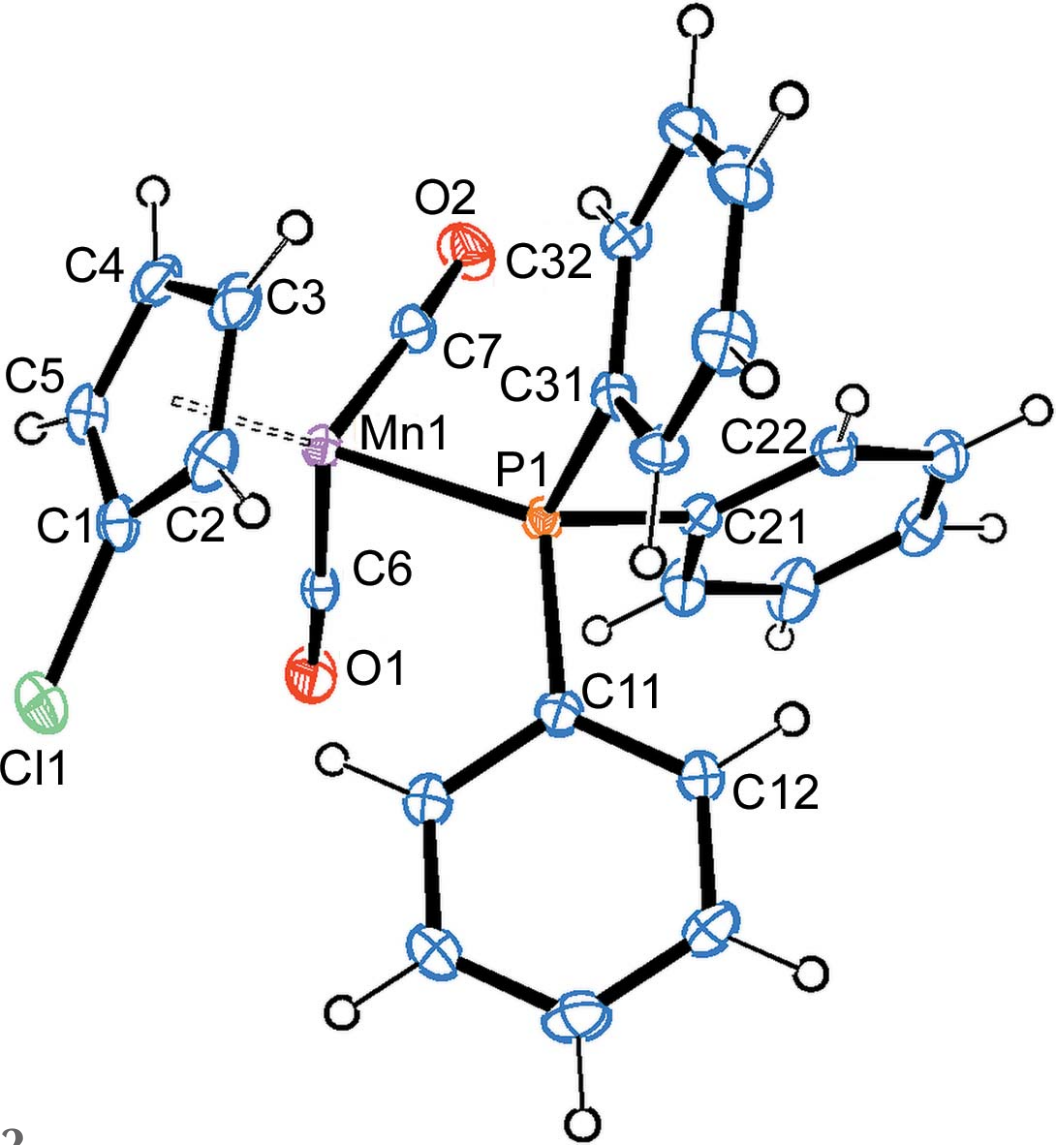
The molecular structure (side view) of com­pound **1b**, with displacement ellipsoids drawn at the 30% probability level.

Compound **1b** crystallizes in the acentric ortho­rhom­bic space group *P*2_1_2_1_2_1_ with one mol­ecule in the asymmetric unit (Fig. 2 ▸). Examination by the program *PLATON* (Spek, 2020 ▸) showed no extra crystallographic symmetry and no sign of racemic twinning. The only ‘mol­ecular’ origin of chirality resides in the PPh_3_ ‘propeller’.

The Mn→P vector is nearly perpendicular to the C—Cl bond (torsion angle C1—Ct—Mn—P1 is 77.6°). The individual bond lengths are nearly identical to those in **1a**; the largest deviation is found for the C—C bonds of the cyclo­penta­dienyl ring, which are slightly (1.5σ) shorter in **1b**. The most important bond parameters can be found in Table 2 ▸.

There is only one intra­molecular C—H⋯Cl hydrogen bond with a length shorter than the sum of the van der Waals radii (H16⋯Cl1). Additionally, there is one weak intra­molecular and three inter­molecular C—H⋯O hydrogen bonds, and one inter­molecular C—H⋯Cl hydrogen bond (Fig. S2 and Table S1 in the supporting information). The Cl atoms always bridge two different H atoms of the same symmetry-related arene ring along the *a* screw axis. Apparently, this inter­action enforces the orientation of this particular arene ring and generates the chirality.

### [(C_5_H_4_ *X*)Mn(CO)_2_(PCy_3_)], *X* = H (2a) and Cl (2b)   

The tri­cyclo­hexyl­phosphane com­pound **2a** was first des­cribed in 1967 (Strohmeier & Müller, 1967 ▸) as part of a study on the π-acceptor strength of phosphane ligands. It was then characterized by IR spectroscopy and elemental analysis. Later on it was shown that its reactivity in hydrogen isotope exchange reactions was more than 15 times greater in com­parison to the PPh_3_ com­pound **1a** (Setkina *et al.*, 1973 ▸). Further spectroscopic characterizations (^13^C and ^31^P NMR) and protonation studies followed soon afterwards (Ginzburg *et al.*, 1974 ▸). The chloro­cyclo­penta­dienyl com­plex **2b** has not been reported before.

We prepared both com­pounds according to Scheme 1 *via* irradiation of the corresponding tricarbonyl com­plexes in the presence of PCy_3_ (tri­cyclo­hexyl­phosphane) in very low yield. Despite long irradiation times, large amounts of the starting material could be recovered. In contrast to **1a**, it was not possible to li­thiate **2a** with *n*-BuLi or *t*-BuLi and chlorinate the presumed inter­mediate lithium com­pound with C_2_Cl_6_ to give **2b**. It was possible, however, to obtain crystals of both com­pounds suitable for X-ray diffraction.

#### Mol­ecular structure of 2a   

**Figure 3:**
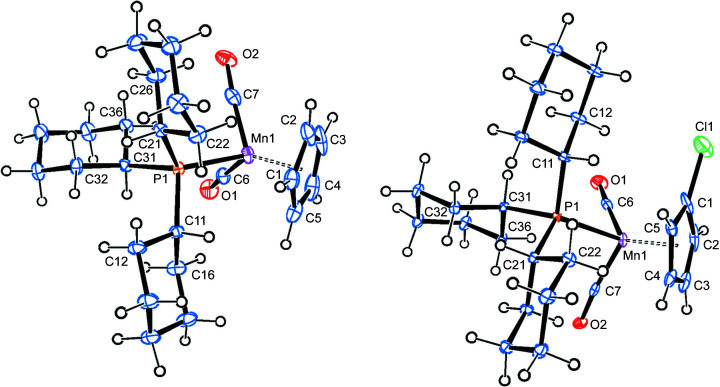
The molecular structures (side view) of com­pounds **2a** (left) and **2b** (right), with displacement ellipsoids drawn at the 30% probability level.

Compound **2a** crystallizes in the monoclinic space group *P*2_1_/*n*, with one mol­ecule in the asymmetric unit (Fig. 3 ▸). The Mn→P vector nearly eclipses a C—H bond of the cyclo­penta­dienyl ring. While the Mn—P bond [2.2661 (7) Å] is significantly longer (50σ) than the average Mn—P bond in **1a**, the Mn—CO bonds are slightly shorter (3–5σ) (Table 2 ▸). The distance from manganese to the cyclo­penta­dienyl centroid is slightly longer (3σ) in **2a** com­pared to **1a**.

There is one intra­molecular and two inter­molecular C—H⋯O hydrogen bonds involving exclusively methyl­ene H atoms of the PCy_3_ ligand and carbonyl atom O1. A packing diagram shows that these inter­actions mainly (although not exclusively) join the individual mol­ecules in the *c* direction (Fig. S3 and Table S1 in the supporting information).

#### Mol­ecular structure of 2b   

Compound **2b** crystallizes in the monoclinic space group *P*2_1_/*c*, with one mol­ecule in the asymmetric unit (Fig. 3 ▸). The Mn→P vector is nearly per­pen­dicular to the C—Cl bond (torsion angle C1—Ct—Mn1—P1 is 78°), with the Mn—P bond [2.2743 (9) Å] being significantly longer (8σ) than in **2a**. The Mn—CO bonds are slightly longer (3σ) than in **2a** and have the same lengths as in **1b**. This is also true for the distance of the Mn atom from the centroid of the cyclo­penta­dienyl ring. More bond parameters can be found in Table 2 ▸.

There are intra­molecular C—H⋯*X* inter­actions involving two methyl­ene H atoms of the PCy_3_ ligand and either the Cl atom or one carbonyl O atom. Additionally, an inter­molecular C—H⋯Cl hydrogen bond joins glide-plane-related mol­ecules along the *b* axis (Fig. S4 and Table S1 in the supporting information).

### [(C_5_H_4_ *X*)Mn(CO)(dppe)], with *X* = H (3a) and Cl (3b)   

The monocarbonyl chelate com­plex **3a** was first prepared by the photochemical reaction of cymantrene with bis­(di­phenyl­phosphan­yl)ethane (dppe) in benzene (*ca* 85% yield after 50 h irradiation), while the same reaction in cyclo­hexane produced the dppe-bridged dinuclear com­plex {[(C_5_H_5_)Mn(CO)_2_]_2_[μ-dppe]} (Nyholm *et al.*, 1963 ▸). Among several studies devoted to spectroscopic characterization and general reactivity, it was found that **3a** had a ninefold decreased kinetic acidity when com­pared to cymantrene (Antonova & Shapiro, 1991 ▸). Compound **3b** has not been reported previously.

Irradiation of THF solutions of the corresponding tricarbonyl com­plexes in the presence of dppe for 7 h yields **3a** and **3b** in modest yields (30–40%), again with substantial recovery of the starting material. Some weak signals in the NMR spectra showed small amounts of other products, most likely dinuclear ones. However, the influence of prolonged reaction times on product yields and distribution was not examined. In contrast to the reactivity of **1b**, it was not possible to deprotonate **3b** [either by lithium diiso­propyl­amide (LDA), lithium tetra­methyl­piperidide (LiTMP) or *t*-BuLi] and introduce more chlorine substituents *via* addition of C_2_Cl_6_. However, again it was possible to obtain crystals suitable for X-ray diffraction for both com­pounds.

#### Mol­ecular structure of 3a   

**Figure 4:**
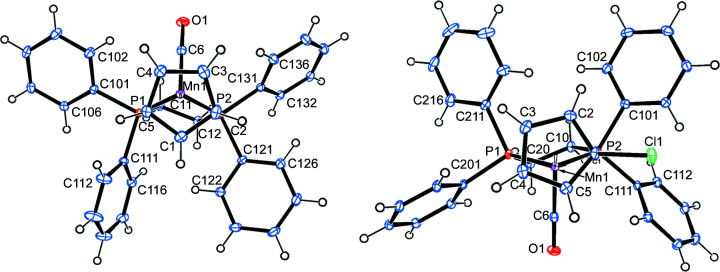
The molecular structures (top views) of com­pounds **3a** (left) and **3b** (right), with displacement ellipsoids drawn at the 30% probability level.

Compound **3a** crystallizes in the monoclinic space group *C*2/*c*, with one mol­ecule in the asymmetric unit. Fig. 4 ▸ shows a top view of the mol­ecular structure. Both Mn→P vectors nearly eclipse two C—H bonds in mutual 1- and 3-positions of the cyclo­penta­dienyl ring, while the Mn→CO vector bis­ects a C—C bond. The Mn—P [2.1968 (4) and 2.1849 (4) Å] and Mn—CO [1.7549 (15) Å] bonds, as well as the distance from manganese to the cyclo­penta­dienyl centroid [1.761 (2) Å], are shorter than for all the above-mentioned com­pounds. At the same time, the C—C bonds of the cyclo­penta­dienyl rings are longer than in the other com­pounds (Table 2 ▸).

There are two inter­molecular hydrogen bonds involving the carbonyl O atom and one methyl­ene H atom of the PCy_3_ ligand or one C—H group of the cyclo­penta­dienyl ring. The packing diagram shows that these inter­actions connect the individual mol­ecules in the *a* direction (Fig. S5 and Table S1 in the supporting information).

#### Mol­ecular structure of 3b   

Compound **3b** crystallizes in the triclinic space group *P* 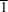, with one mol­ecule in the asymmetric unit (Fig. 4 ▸). The Mn→P2 vector bis­ects the C—C bond next to the chlorine substituent, while the Mn→P1 and Mn→CO vectors nearly eclipse two C—H bonds in the 2- and 4-positions. The Mn—P bond lengths [2.1961 (5) and 2.2024 (5) Å] are significantly different from each other (by 12σ) and slightly longer than in **3a**. The same holds for the relative distances between manganese and the cyclo­penta­dienyl centroids, while the Mn—CO bonds are virtually identical (Table 2 ▸). It is worth noting the near perpendicular orientation of one arene ring (C201–C206) relative to the cyclo­penta­dienyl ring (inter­planar angle 86.0°). This leads to a rather close approach of arene H atom H206 to cyclo­penta­dienyl atom H4 (2.375 Å).

There is one inter­molecular C—H⋯Cl hydrogen bond involving an arene H atom, which joins the individual mol­ecules in the *b* direction. The carbonyl O atom joins two mol­ecules in the *a* direction, employing one arene H atom and one cyclo­penta­dienyl H atom each (Fig. S6 and Table S1 in the supporting information).

## Comparison of the structures and conclusion   

The introduction of a chlorine substituent in the cyclopentadienyl ring leads to a slight increase in the Mn—Ct and Mn—P distances for all the title phosphanes, while both the Mn—CO and the C—O bonds are only affected in the PCy_3_ system, where a substantial elongation occurs. When com­paring the two triads with different phosphanes, the Mn—Ct (Ct des­cribes the centroid of the cyclo­penta­dienyl ring) and Mn—P distances show a slight increase in the order **3**→**1**→**2**. The C—O bonds follow the trend **1** ≃ **2** < **3** and the C—Cl bonds follow the trend **2b** < **1b** ≃ **3b**. The average C—C bond lengths are the same within 2σ for all six com­pounds. Comparison with the PPh_2_CH_2_Ph com­pound GIXRIO and the ferrocenylbis­phosphane chelate com­pound EFUHAO shows more similarities with the PPh_3_ com­plexes **1** than with the dppe chelates **3**. The tendency of the Mn—P bonds to eclipse one cyclo­penta­dienyl C—H bond is obvious in all the com­pounds. In all the chloro com­pounds, the Mn—P bonds avoid being eclipsed with the C—Cl bond of the cyclopentadienyl ring.

Apparently, the introduction of one chlorine substituent has only a small influence on the bond lengths, despite the relatively large effect on the spectroscopic data. Steric hindrance within the phosphanes seems to be of greater importance for the bond parameters than the differences in electronic effects. However, the presence of chlorine in the cyclo­penta­dienyl ring leads to additional lattice stabilization *via* the formation of C—H⋯Cl hydrogen bonds.

## Supplementary Material

Crystal structure: contains datablock(s) compd1a, compd1b, compd2a, compd2b, compd3a, compd3b, global. DOI: 10.1107/S2053229621009177/dv3012sup1.cif

Structure factors: contains datablock(s) compd1a. DOI: 10.1107/S2053229621009177/dv3012compd1asup2.hkl

Structure factors: contains datablock(s) compd1b. DOI: 10.1107/S2053229621009177/dv3012compd1bsup3.hkl

Structure factors: contains datablock(s) compd2a. DOI: 10.1107/S2053229621009177/dv3012compd2asup4.hkl

Structure factors: contains datablock(s) compd2b. DOI: 10.1107/S2053229621009177/dv3012compd2bsup5.hkl

Structure factors: contains datablock(s) compd3a. DOI: 10.1107/S2053229621009177/dv3012compd3asup6.hkl

Structure factors: contains datablock(s) compd3b. DOI: 10.1107/S2053229621009177/dv3012compd3bsup7.hkl

Additional figures and table. DOI: 10.1107/S2053229621009177/dv3012sup8.pdf

CCDC references: 2107491, 2107490, 2107489, 2107488, 2107487, 2107486

## Figures and Tables

**Table:** Experiments were carried out with Mo *K*α radiation using a Bruker D8 Venture (for **1a**, **2b**, **3a** and **3b**) or an Oxford Diffraction KM4 Xcalibur2 (for **1b** and **2a**) diffractometer.

	**1a**	**1b**	**2a**
Crystal data			
Chemical formula	[Mn(C_5_H_5_)(C_18_H_15_P)(CO)_2_]	[Mn(C_5_H_4_Cl)(C_18_H_15_P)(CO)_2_]	[Mn(C_5_H_5_)(C_18_H_33_P)(CO)_2_]
*M*_r_	438.32	472.76	456.46
Crystal system, space group	Monoclinic, *P*2_1_/*n*	Orthorhombic, *P*2_1_2_1_2_1_	Monoclinic, *P*2_1_/*n*
Temperature (K)	110	173	173
*a*, *b*, *c* (Å)	7.6736 (4), 15.7356 (8), 33.912 (2)	7.6519 (3), 16.4786 (7), 17.0971 (7)	9.8938 (5), 13.6564 (5), 17.9372 (9)
α, β, γ (°)	90, 95.942 (2), 90	90, 90, 90	90, 105.676 (5), 90
*V* (Å^3^)	4072.9 (4)	2155.82 (15)	2333.42 (19)
*Z*	8	4	4
μ (mm^−1^)	0.75	0.83	0.65
Crystal size (mm)	0.05 × 0.05 × 0.03	0.34 × 0.14 × 0.10	0.33 × 0.23 × 0.14

Data collection			
Absorption correction	Multi-scan (*SADABS*; Krause *et al.*, 2015 ▸)	Multi-scan (*CrysAlis PRO*; Agilent, 2014 ▸)	Multi-scan (*CrysAlis PRO*; Agilent, 2014 ▸)
*T*_min_, *T*_max_	0.709, 0.746	0.892, 1	0.990, 1
No. of measured, independent and observed [*I* > 2σ(*I*)] reflections	64715, 8968, 7809	14524, 4900, 4297	15861, 5333, 3805
*R*_int_	0.048	0.045	0.054
(sin θ/λ)_max_ (Å^−1^)	0.641	0.649	0.649

Refinement			
*R*[*F*^2^ > 2σ(*F* ^2^)], *wR*(*F* ^2^), *S*	0.038, 0.090, 1.07	0.038, 0.082, 1.04	0.045, 0.119, 1.03
No. of reflections	8968	4900	5333
No. of parameters	523	271	262
No. of restraints	0	0	1
H-atom treatment	H-atom parameters constrained	H-atom parameters constrained	H-atom parameters constrained
Δρ_max_, Δρ_min_ (e Å^−3^)	0.55, −0.65	0.39, −0.29	0.67, −0.47
Absolute structure	–	Flack *x* determined using 1609 quotients [(*I* ^+^) − (*I* ^−^)]/[(*I* ^+^) + (*I* ^−^)] (Parsons *et al.*, 2013 ▸)	–
Absolute structure parameter	–	−0.038 (12)	–

**Table:** 

	**2b**	**3a**	**3b**
Crystal data			
Chemical formula	[Mn(C_5_H_4_Cl)(C_18_H_33_P)(CO)_2_]	[Mn(C_5_H_5_)(C_26_H_24_P_2_)(CO)]	[Mn(C_5_H_4_Cl)(C_26_H_24_P_2_)(CO)]
*M*_r_	490.90	546.43	580.87
Crystal system, space group	Monoclinic, *P*2_1_/*c*	Monoclinic, *C*2/*c*	Triclinic, *P*\overline{1}
Temperature (K)	100	100	100
*a*, *b*, *c* (Å)	9.6649 (3), 13.9301 (4), 17.9790 (6)	29.0323 (7), 8.9592 (2), 26.4794 (7)	8.5739 (5), 11.5697 (8), 14.3909 (9)
α, β, γ (°)	90, 103.835 (1), 90	90, 122.159 (1), 90	90.584 (2), 91.958 (2), 110.490 (2)
*V* (Å^3^)	2350.34 (13)	5830.7 (3)	1336.07 (15)
*Z*	4	8	2
μ (mm^−1^)	0.76	0.58	0.74
Crystal size (mm)	0.10 × 0.08 × 0.06	0.10 × 0.08 × 0.07	0.08 × 0.06 × 0.03

Data collection			
Absorption correction	Multi-scan (*SADABS*; Krause *et al.*, 2015 ▸)	Multi-scan (*SADABS*; Krause *et al.*, 2015 ▸)	Multi-scan (*SADABS*; Krause *et al.*, 2015 ▸)
*T*_min_, *T*_max_	0.704, 0.745	0.719, 0.746	0.702, 0.745
No. of measured, independent and observed [*I* > 2σ(*I*)] reflections	31339, 4809, 4098	70409, 6696, 6042	24803, 5453, 4783
*R*_int_	0.039	0.032	0.035
(sin θ/λ)_max_ (Å^−1^)	0.625	0.650	0.626

Refinement			
*R*[*F*^2^ > 2σ(*F* ^2^)], *wR*(*F* ^2^), *S*	0.049, 0.149, 1.06	0.030, 0.081, 1.09	0.027, 0.063, 1.04
No. of reflections	4809	6696	5453
No. of parameters	271	325	334
No. of restraints	1	0	0
H-atom treatment	H-atom parameters constrained	H-atom parameters constrained	H-atom parameters constrained
Δρ_max_, Δρ_min_ (e Å^−3^)	0.73, −1.64	0.39, −0.36	0.39, −0.31

Computer programs: *APEX2* (Bruker, 2011 ▸), *CrysAlis PRO* (Agilent, 2014 ▸), *SAINT* (Bruker, 2011 ▸), *SHELXT2014* (Sheldrick, 2015*a* ▸), *SHELXT2018* (Sheldrick, 2015*a* ▸) and *SHELXL2018* (Sheldrick, 2015*b* ▸).

**Table 2:** Important bond parameters of **1**–**3** in com­parison with two related literature com­pounds Ct is the centroid of the cyclo­penta­dienyl ring, (C—C)_av_ is the average C—C bond length within the cyclo­penta­dienyl ring and C_*x*_—Ct—Mn—P is the smallest torsion angle involving a cyclo­penta­dienyl C—H (**1a**, **2a** and **3a**) or C—Cl bond.

Distance/angle	**1a** mol. *A*/ mol. *B*	**1b**	**2a**	**2b**	**3a**	**3b**	GIXRIO	EFUHAO
Mn—Ct	1.777 (1)/1.778 (1)	1.786 (2)	1.781 (1)	1.786 (1)	1.761 (2)	1.768 (1)	1.773 (2)	1.769 (9)
Mn—P	2.2256 (8)/2.2423 (8)	2.2403 (10)	2.2661 (7)	2.2743 (9)	2.1968 (4), 2.1849 (4)	2.1961 (5), 2.2024 (5)	2.2198 (2)	2.244 (3), 2.241 (3)
Mn—CO	1.769 (2), 1.776 (2)/1.777 (2), 1.767 (2)	1.772 (4), 1.770 (3)	1.763 (2), 1.761 (2)	1.774 (3), 1.773 (3)	1.756 (5)	1.755 (2)	1.755 (5)	1.769 (9)
C—O	1.165 (3), 1.162 (3)/1.162 (3), 1.160 (3)	1.155 (5), 1.164 (4)	1.162 (3), 1.174 (4)	1.161 (4), 1.162 (4)	1.174 (6)	1.172 (2)	1.161 (7), 1.165 (6)	1.15 (1)
C—Cl	–	1.730 (4)	–	1.674 (4)	–	1.737 (2)	–	–
(C—C)_av_	1.416 (3)/1.415 (4)	1.408 (5)	1.408 (4)	1.411 (6)	1.422 (5)	1.416 (2)	1.395 (7)	1.41 (2)
C_Cl_—Ct—Mn—P	–	77.6	–	78.0	–	36.3, 156.0	–	–
C_H_—Ct—Mn—P	13.0/8.1	20.5	12.3	7.2	14.1, 13.4	36.0, 12.1	10.2	4.4, 13.6
