# Supplementary material for: Crystal and mol­ecular structures of some phosphane-substituted cymantrenes [(C5H4 X)Mn(CO)LL′] (X = H or Cl, L = CO, L′ = PPh3 or PCy3, and LL’ = Ph2PCH2CH2PPh2)
Source: Acta Crystallogr C Struct Chem. 2021 Sep 27;77(Pt 10):633–40. doi: 10.1107/S2053229621009177 (PMC8491095; doi:10.1107/S2053229621009177)
Supplement: Supplementary file 8 [file c-77-00633-sup8.pdf]

STRUCTURAL CHEMISTRY

Volume 77 (2021)

Supporting information for article:

Crystal and molecular structures of some phosphane-substituted cymantrenes [(C5H4*X*)Mn(CO)*LL'*] (*X* = H or Cl, *L* = CO, *L'* = PPh3 or PCy3, and *LL*' = Ph2PCH2CH2PPh2)

Karlheinz Sünkel and Christian Klein-Hessling

S1. Packing Diagrams

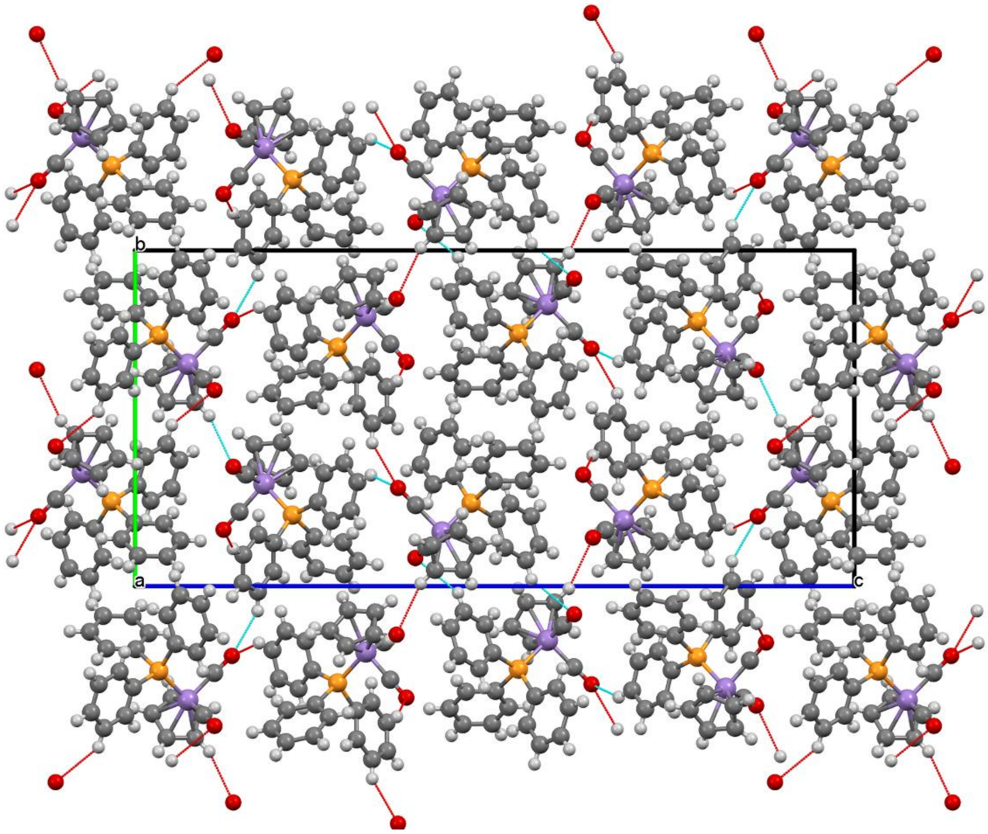

Figure S1 Packing diagram (MERCURY) of compound **1a** watched along the *a* axis. Red and blue lines show C–H…O hydrogen bonds (colour coding as defined by mercury; red are “hanging contacts” and blue are “not-hanging contacts”).

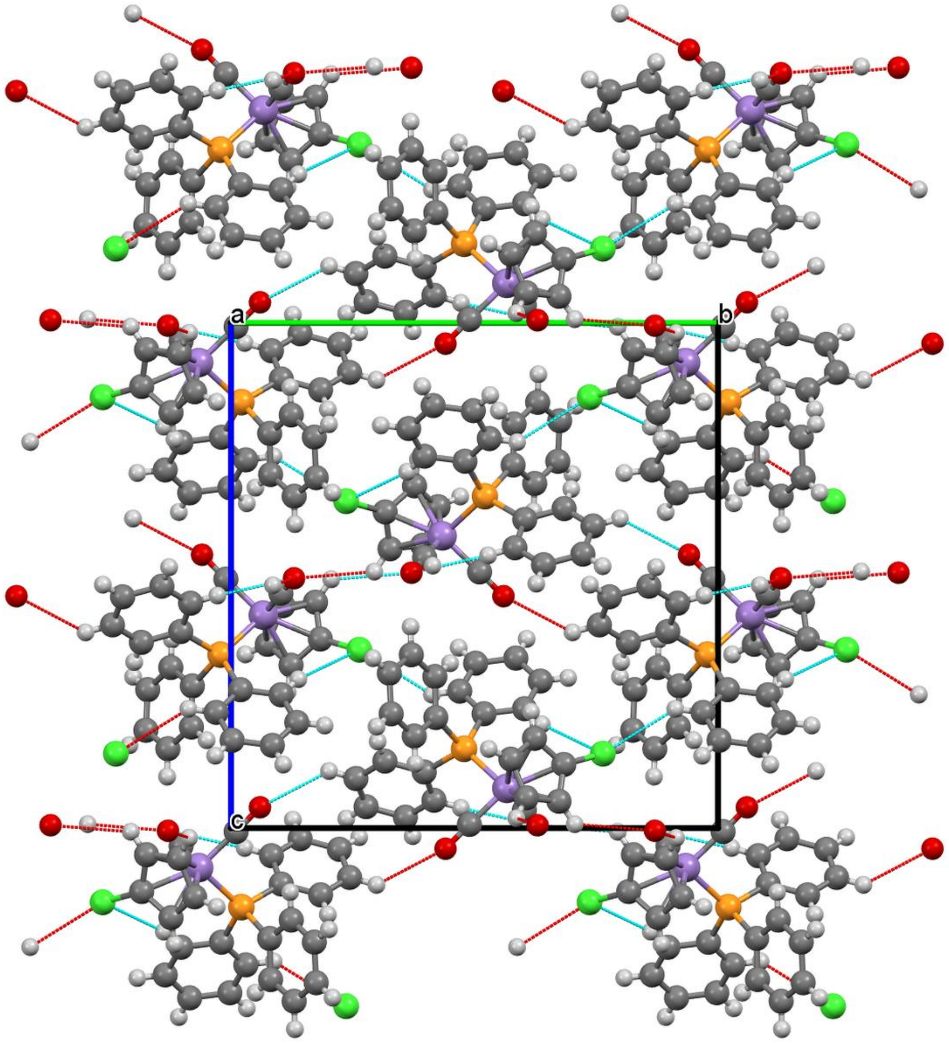

Figure S2 Packing diagram (MERCURY) of compound **1b** watched along the *a* axis. Red and blue lines show C–H…Cl and C–H…O hydrogen bonds (colour coding as defined by mercury; red are “hanging contacts” and blue are “not-hanging contacts”)

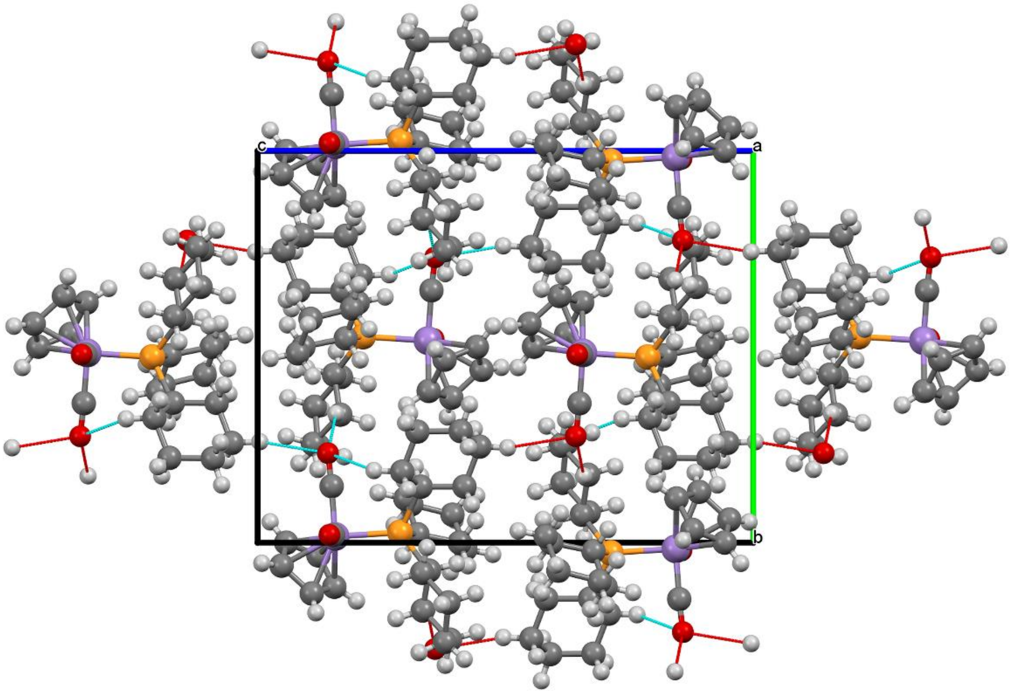

Figure S3 Packing diagram (MERCURY) of compound **2a** watched along the *a* axis. Red and blue lines show C–H…O hydrogen bonds (colour coding as defined by mercury; red are “hanging contacts” and blue are “not-hanging contacts”)

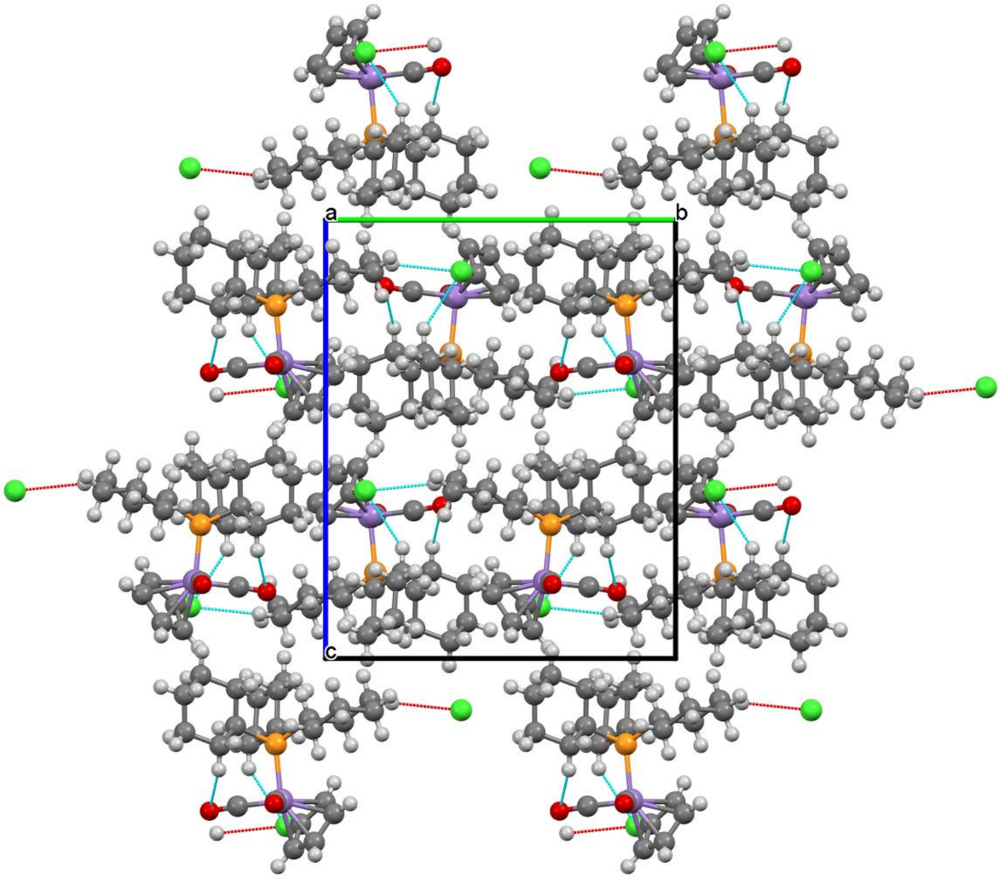

Figure S4 Packing diagram (MERCURY) of compound **2b** watched along the *a* axis. Red and blue lines show C–H…O and C–H…Cl hydrogen bridges (colour coding as defined by mercury; red are "hanging contacts" and blue are "not-hanging contacts")

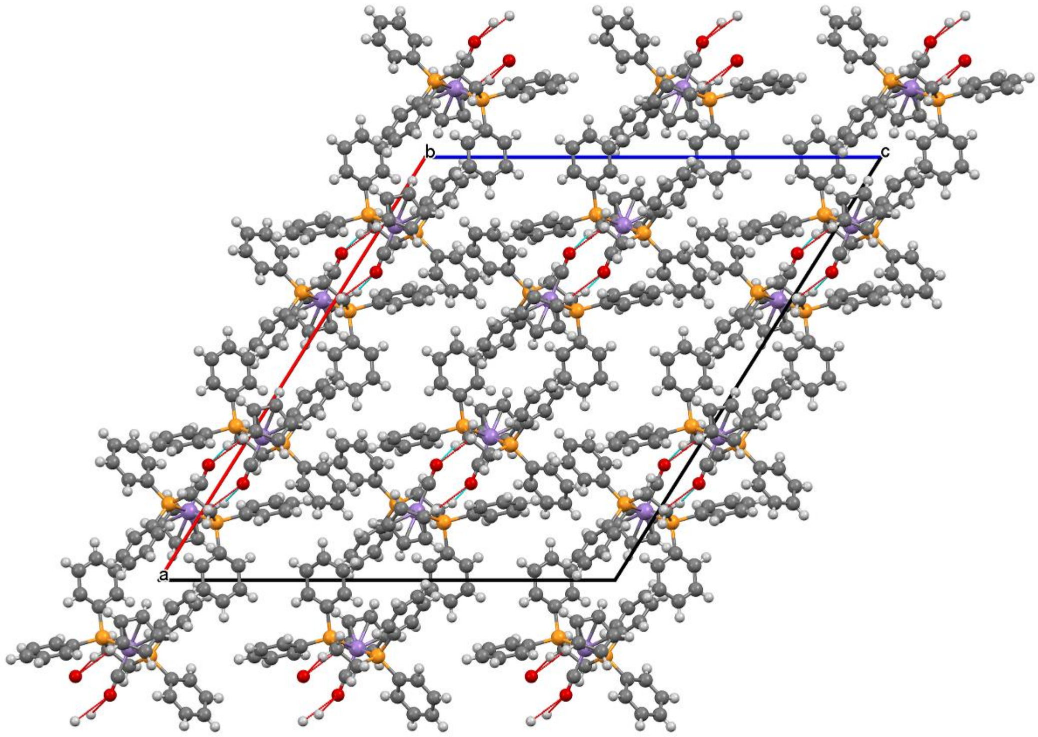

Figure S5 Packing diagram (MERCURY) of compound **3a** watched along the *b* axis. Red and blue lines show C–H…O hydrogen bonds (colour coding as defined by mercury; red are “hanging contacts” and blue are “not-hanging contacts”)

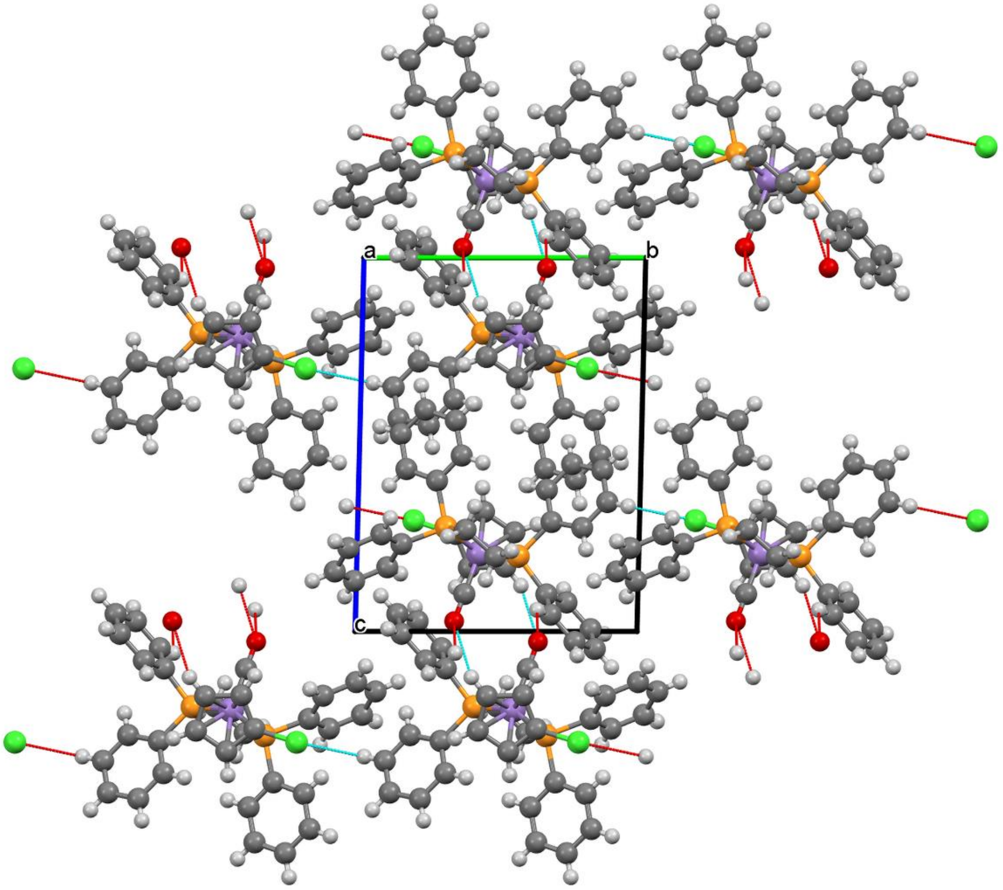

Figure S6 Packing diagram (MERCURY) of compound **3b** watched along the *a* axis. Red and blue lines show C–H…O and C–H…Cl hydrogen bonds (colour coding as defined by mercury; red are "hanging contacts" and blue are "not-hanging contacts")

Table S1 Hydrogen bonds

Compound	Atom Pair	Distance [Å]	Symmetry code
**1a**	H103…O11	2.577	x-1, y, z
	O12…H23	2.677	1-x, -y, 1-z
	H105…O22	2.528	1-x, 1-y, 1-z
	H124…O22	2.616	x- ½, ½ -y, ½ +z
	O21…H205	2.686	2-x, -y, 1-z
**1b**	H16…Cl1	2.649	x, y, z
	Cl1…H12	2.999	1-x, y- ½ , ½-z
	H26…O1	2.746	x, y, z
	O1…H4	2.761	x-1,y,z
	O1…H5	2.750	x- ½ , ½-y, 1-z
	H23…O2	2.770	x- ½ ,1.5-y, 1-z
**2a**	H36B…O1	2.538	x, y, z
	H22A…O1	2.584	1.5-x, y- ½ , ½ -z
	H33B…O1	2.626	x- ½ ,1.5-y, z- ½
**2b**	H12A…Cl1	2.835	x, y, z
	H36A…O1	2.694	x, y, z
	Cl1…H23B	2.821	1-x, y- ½ , ½ -z
**3a**	O1…H3	2.647	½ -x, ½ -y, 1-z
	O1…H12A	2.713	½ -x, 1.5-y, 1-z
**3b**	Cl1…H215	2.887	x, y-1, z
	H202…O1	2.651	1-x, 1-y, 2-z
	H4…O1	2.355	2-x, 1-y, 2-z
